# Changes in Bacterial Community Structure and Enriched Functional Bacteria Associated With Turfgrass Monoculture

**DOI:** 10.3389/fbioe.2020.530067

**Published:** 2021-01-15

**Authors:** Chang-Wook Jeon, Da-Ran Kim, Eun-Ji Bae, Youn-Sig Kwak

**Affiliations:** ^1^Dvision of Applied Life Science (BK21 Plus) and Research Institute of Life Sciences, Gyeongsang National University, Jinju, South Korea; ^2^Forest Biomaterials Research Center, National Institute of Forest Science, Jinju, South Korea; ^3^Department of Plant Medicine, Institute of Agriculture & Life Science, Gyeongsang National University, Jinju, South Korea

**Keywords:** antifungal activity, *Burkholderia*, monoculture, plant growth promotion, *Streptomyces*, turfgrass

## Abstract

There is increasing attention being paid to utilizing microbial communities to improve plant health while reducing management inputs. Thus, the objectives of this research were to assess changes in the rhizosphere bacterial community structure associated with long-term turfgrass monoculture and to demonstrate the feasibility of using functional bacteria as beneficial biocontrol agents. Large patch disease, caused by the fungal pathogen *Rhizoctonia solani* AG2-2, is a significant threat to turfgrass cultivation. Rhizosphere samples were collected from 2-, 13- and 25-year turfgrass (*Zoysia japonica*) monocultures. The 13-year monoculture field had a higher pathogen population density than both the 2- and 25-year monoculture fields. Analyses of the rhizosphere bacterial communities revealed that *Streptomyces* was dominant in the 2-year field and *Burkholderia* was enriched in the 25-year field. Based on the culturable rhizosphere bacteria, *Streptomyces neyagawaensis* J6 and *Burkholderia vietnamiensis* J10 were obtained from the 2- and 25-year fields, respectively. Application of *S. neyagawaensis* J6 and *B. vietnamiensis* J10 led to excellent inhibition of large patch disease as well as enhanced tolerance against drought and temperature stresses. The results showed that the selected bacteria could be developed as biocontrol and abiotic stress tolerance agents for turfgrass cultivation.

## Introduction

Turfgrass is considered as one of the most important irrigated plants worldwide. It is significantly involved in the regulation of carbon sequestration, soil erosion control, and cooling, while also supporting nutrient cycles and leading with recreational value for human activities (Beard, 1972; Simmons et al., 2011). Soil-borne fungal pathogens are the primary cause of the wide range of diseases observed in turfgrass (Asano et al., 2010; Obasa et al., 2012). Among the pathogens, *Rhizoctonia solani* AG2-2, which causes large patch disease, is the most problematic due to its ability to infect the leaf blades, sheath, and crown of turfgrass. Infected turfgrass exhibits yellow discoloration, which is an early symptom of an outbreak of large patch disease (Couch et al., 1990). This disease is the greatest threat to turfgrass plantings throughout the world and can cause disease repeatedly every year in the same location (Smiley et al., 2005). The onset of infection occurs during autumn before turfgrass enters winter dormancy. The most effective preventative measure involves regular application of fungicides (such as azoxystrobin, flutolanil, or tebuconazole) with a typical split application process in autumn and the following spring (Vincelli and Munshaw, 2014). This repeated use of fungicides not only entails significant expense (van Bruggen and Arneson, 1984) but also raises concerns about environmental pollution and the emergence of fungicide-resistant pathogens. Therefore, the use of beneficial microorganisms to counter the effects of the disease is considered a potentially eco-friendly and cost-effective option (Zhang et al., 2019).

Plants' ability to absorb nutrients can be improved through symbiosis with rhizosphere microorganisms (Raghothama and Karthikeyan, 2005). Root exudates, which can contain >20% of the carbon assimilated by photosynthesis and other small metabolites, are secreted through the plant root system into the rhizosphere (Jones et al., 2009). Therefore, the rhizosphere is a nutrient-rich zone for soil microorganisms. Among the various components of root exudates, aromatic organic acids are consumed by certain rhizobacteria (Zhalnina et al., 2018). This is also reflected at the genome level, with rhizosphere-associated bacterial genomes being enriched for carbohydrate metabolism genes (Levy et al., 2018). Upon surviving and sucessfully colonizing the rhizosphere, certain microbes offer protection (against both biotic and abiotic stresses) for the plants on which they thrive as well as enhancing the absorption of nutrients such as nitrogen and phosphorus (Richardson, 2001; Tang et al., 2019). Additionally, plant genotypes, developmental stage, and soil types can all exert an influence on the diversity and composition of the rhizosphere microbial community (Marasco et al., 2018; Poudel et al., 2019). In monoculture system, wheat take-all disease (TAD) is a well-known disease model in addition to its biocontrol agent *Pseudomonas* spp., which was isolated from the wheat rhizosphere (Raaijmakers and Weller, 1998). Similarly, Cha et al. (2016) reported that *Streptomyces* sp. S4-7 prevented Fusarium wilt disease during strawberry cultivation; this strain was isolated from the rhizosphere of strawberries based on the results of massive pyrosequencing and microbial community analyses. *Streptomyces* sp. S8 (Jeon et al., 2019) has been reported to significantly reduce the occurrence of turfgrass soil-borne disease. A common feature of the above mentioned beneficial strains is that they were isolated from the rhizosphere of their relevant host plants. This indicates that the soil, especially the rhizosphere, is the home of numerous microbes and that certain beneficial microbes have strong positive interactions with plants. However, information on the effects of long-term monoculture on the structure of the microbial communities is still insufficient. Thus, the purpose of this study was to assess changes in the microbial community structure associated with turfgrass monoculture of various durations and to demonstrate the feasibility of using selected bacteria as biocontrol agents.

## Results

### Physicochemical Properties of the Soils

The three investigated *Z. japonica* monoculture fields were located in the same area in Jangseong-gun, Republic of Korea (GPS locations, 2-year field: N35°11, E126°40; 13-year field: N35°11, E126° 40; 25-year field: N35°11, E126 ^o^40). Among the analyzed chemical and physical properties of the soils, bulk density and water content were higher in the 25-year field than the 2- and 13-year fields. In contrast, the 25-year field had the lowest soil porosity and soil pH. There were no significant differences in electrical conductivity, total nitrogen, organic matter, available P_2_O_5_, or ion levels among the 2-, 13-, and 25-year soils (Supplementary Tables 1, 2).

### Rhizosphere Microbial Communities and Density of Large Patch Disease Causing Pathogen

**Figure 1:**
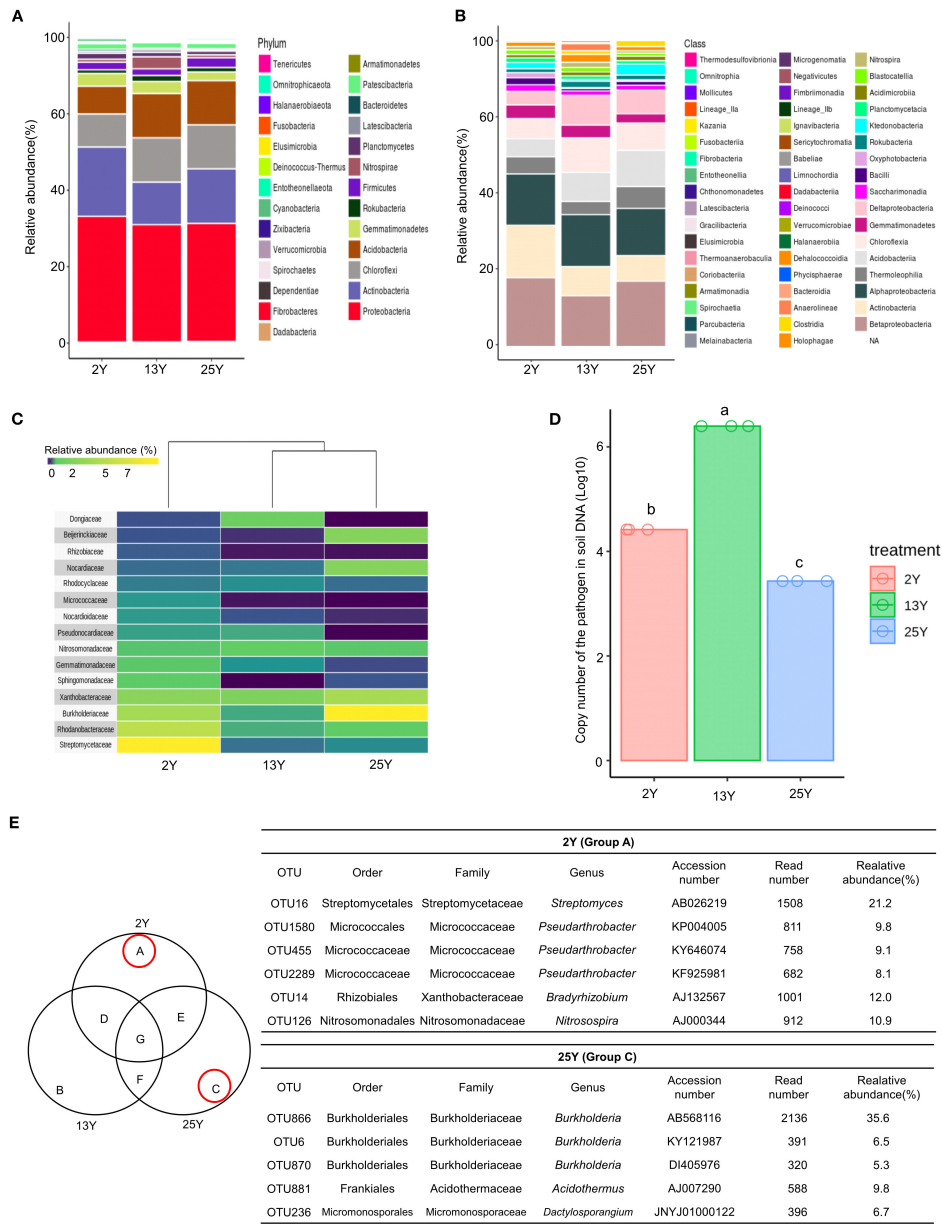
Microbial diversity in rhizosphere of 2-, 13-, and 25-year turfgrass monoculture fields (*n* = 3). Taxonomic assignment was conducted using the Silva database (http://www.arb-silva.de/) and a cutoff of 97% similarity. **(A)** Phylum level **(B)** Class level. **(C)** Heatmap showing hierarchical clustering of bacterial communities based on 16S rRNA. Bluish-violet to yellow indicates low to high OTU abundance at the family level. **(D)** Population density of the large patch pathogen *R. solani* in the soil of different turfgrass fields, **(E)** Venn diagram of numbers of unique and shared OTUs in the rhizosphere samples. Dominant microbes in the 2- and 25-year rhizospheres are identified as groups A and C, respectively.

Rhizosphere soil samples (*n* = 3 per field) were collected per fields that had been continuously cultivated for 2, 13, and 25 years. Bacterial DNA sequences (Supplementary Table 3) were analyzed to identify the number of operational taxonomic units (OTUs), which was found to be 5,939 (Supplementary Table 4). At the phylum level, Proteobacteria was the most dominant phylum across all three soils (2-, 13-, and 25-year soils); additionally, the 2-year soil had a high level of Actinobacteria (17.7%), whereas the 13- and 25-year soils had lower levels (8.2 and 12.8%, respectively) (Figure 1A). At the class level, Betaproteobacteria was the most dominant class in the 2- and 25-year soils, whereas the 13-year soil was dominated by Alphaproteobacteria. Actinobacteria accounted for the highest proportion in the 2-year soil (Figure 1B). After conducting heatmap analyses and hierarchical cluster analyses using Euclidean distance at the family level, a clear distinction was identified between the 2- and 13-year soils and the 25-year soil. The family *Streptomycetaceae* was the most dominant bacteria in the 2-year soil, while *Burkholderiaceae* was the most dominant in the 25-year soil. However, regarding the 13-year soil, no dominant bacteria (accounting for >7%) was detected (Figure 1C). Regarding the population density of the large patch disease pathogen, *R. solani* AG2-2, in the soil DNA samples, the copy number was 10^6^ per 100 ng of soil DNA in the 13-year soil, whereas it was 10^4^ and 10^3^ in the 2- and 25-year soils, respectively (Figure 1D). The Venn diagram (Figure 1E) shows the unique bacteria associated with the 2- and 25-year soils, which are labeled groups A and C, respectively; *Streptomyces* (OTU16) accounted for the highest relative abundance (21.2%) in group A and *Burkholderia* (OTU866) accounted for the highest relative abundance (35.6%) in group C. Thus, it was speculated that *Streptomyces* and *Burkholderia* served as key microbes in the turfgrass soils that had undergone 2 and 25 years of monoculture, respectively.

### Antifungal Activity of the Selected Strains and Inhibition of Large Patch Disease

**Figure 2:**
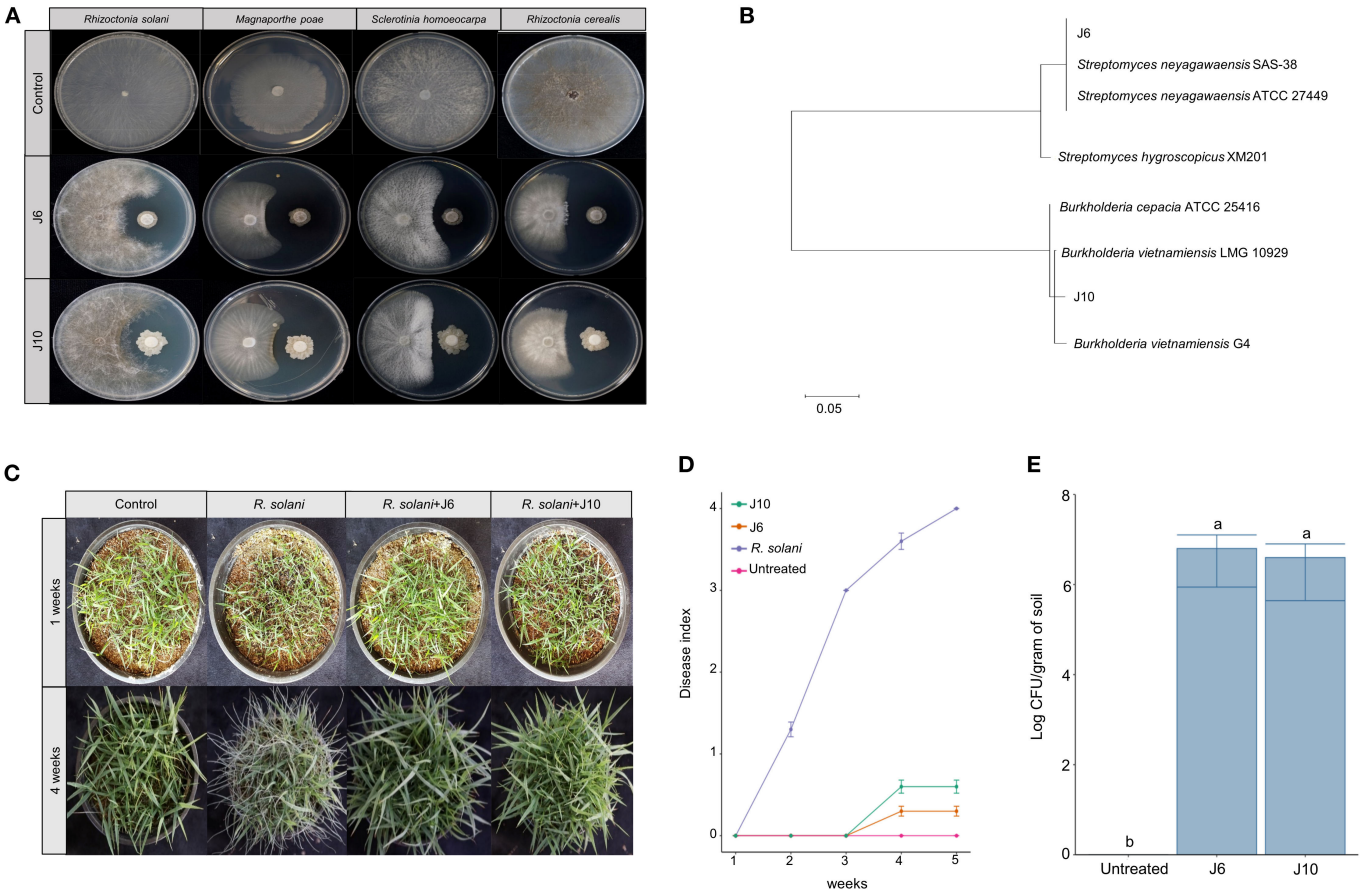
Effectiveness of inhibition of large patch pathogen *R. solani* by *Streptomyces* sp. J6 and *Burkholderia vietnamiensis* J10 in *Z. japonica*. **(A)** Inhibition tests of *Streptomyces* sp. J6 and *B. vietnamiensis* J10 against large patch, summer patch, dollar spot, and spring dead spot on potato dextrose peptone (PDK) agar. **(B)** Phylogenetic tree of J6 and J10 based on 16S rRNA. Sequences were compared by multiple alignment analysis using the maximum likelihood method in MEGA 7. **(C)** Pot assays of inhibition of large patch disease by J6 and J10 (10^4^ cfu/g in 5 g plastic pots) observed after 4 weeks. **(D)** Large patch disease severity (*n* = 10). Disease index values of 0 to 4 represent 0–20%, 21–40%, 41–60%, 61–80%, and 81–100%, respectively. **(E)** Population density of J6 and J10 in the turfgrass rhizospheres. The bacterial densities were calculated by the dilution plate method on selective PDK medium (hygromycin 80 μg/mL or rifampicin 100 μg/mL; *n* = 10). Different letters indicate a significant difference based on Duncan's multiple range test at *P* ≤ 0.05.

Using a culture-dependent approach, 892 bacterial strains were isolated from the 2-, 13-, and 25-year monoculture fields (i.e., 298, 296, and 298 bacterial strains, respectively). The isolated bacteria were screened for antifungal activity against the large patch pathogen, *R. solani*. Ultimately, two strains of antifungal bacteria (i.e., J6 and J10) were obtained. These strains were not only effective against *R. solani* but also against other turfgrass pathogens that cause summer patch, dollar spot, and spring dead spot diseases (Figure 2A, Supplementary Table 6). The J6 and J10 strains were identified using their 16S rRNA sequences. The J6 strain was identified as *Streptomyces* sp. and the J10 strain was identified as *Burkholderia vietnamiensis*, with 99% similarity (Figure 2B). Interestingly, the *Streptomyces* sp. J6 strain was isolated from a rhizosphere of the 2-year monoculture field and the *B. vietnamiensis* J10 strain was obtained from a rhizosphere of the 25-year field. The 16S rRNA sequences of J10 (MT573529.1) and J6 (FJ999671.1) were matched with 100% identity of OTU866 and OTU16, respectively. This finding supports the pyrosequencing results showing that *Streptomyces* and *Burkholderia* represented unique and dominant OTUs in the 2- and 25-year fields, respectively. Subsequently, the two strains' abilities to inhibit large patch disease and colonize the rhizosphere were investigated using pot assays (*Z. japonica*). Both strains significantly reduced the damage inflicted by *R. solani* (Figures 2C,D). The population densities of both strains in the *Z. japonica* rhizosphere was >10^6^ cfu/g of soil (Figure 2E).

### Abiotic Stress Alleviation by the J6 and J10 Strains

**Figure 3:**
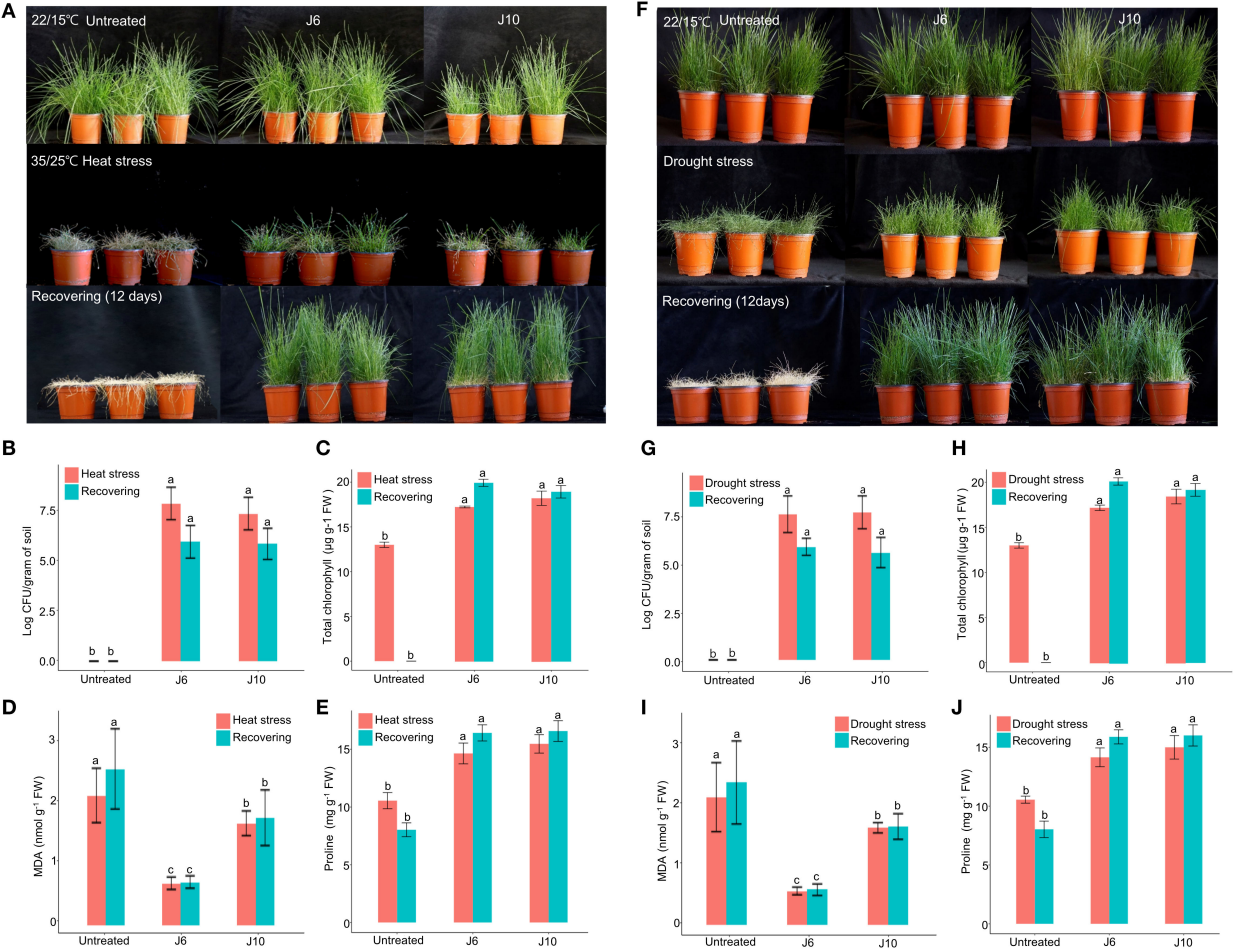
Alleviation of heat and drought stress effects by *Streptomyces* sp. J6 and *Burkholderia vietnamiensis* J10 strains in *A. stolonifera*. **(A)** Application of heat stress and on completion recovery response. **(B)** Population density of J6 and J10 in turfgrass rhizospheres. **(C)** Total chlorophyll concentration. **(D)** Malondialdehyde (MDA) concentration. **(E)** Proline concentration. **(F)** drought stress application and recovery response in *A. stolonifera*. **(G)** Population density of J6 and J10 in the rhizospheres. **(H)** Total chlorophyll concentration in *A. stolonifera*. **(I)** Malondialdehyde (MDA) concentration and **(J)** proline concentration. Different letters indicate a significant difference based on Duncan's multiple range test at *P* ≤ 0.05.

For cool-season turfgrass cultivars, heat and drought are the most destructive abiotic stresses, especially during summer. To evaluate the alleviation of the effects of abiotic stresses by the J6 and J10 strains, a cool-season turfgrass cultivar (*Agrostis stolonifera*) was exposed to heat stress and drought stress separately. Heat stress for 10 days caused more damage in the control turfgrass compared to the J6- and J10-treated turfgrasses (whose growth and development remained largely unaffected by the heat stress). Subsequently, in spite of a recovery period of 12 days, the control *A. stolonifera* withered, but the J6- and J10-treated *A. stolonifera* recovered (Figure 3A). After both the heat stress period and the recovery period, both the J6 and J10 strains colonized at high population densities of up to 10^6^ cfu/g of soil (Figure 3B). This result indicates that both strains stably colonized the rhizospheres even under severe abiotic stress. Total chlorophyll, malondialdehyde (MDA, the breakdown product of unsaturated fatty acids), and proline concentrations in the *A. stolonifera* were measured after both the heat stress and recovery periods. Both of MDA and proline can accumulate in grass leaves under stress. Under heat stress, the total chlorophyll concentration was 13, 18, and 19 μg/g of fresh weight (g^−1^ FW) for untreated, J6-treated, and J10-treated turfgrasses, respectively. After 12 days of recovery, the chlorophyll concentration was 19.5 and 19 μg g^−1^ FW in the J6- and J10-treated *A. stolonifera*, but no chlorophyll was detected in the untreated *A. stolonifera* (Figure 3C). The MDA concentration was significantly lower in the J6- and J10-treated *A. stolonifera* compared to the untreated control *A. stolonifera* both under heat stress and after the recovery period (Figure 3D). Regarding the proline concentration, the J6- and J10-treated *A. stolonifera* had significantly higher levels than the untreated control *A. stolonifera* (Figure 3E).

All drought stress results resembled the heat stress results. After inducing drought stress for 10 days, recovery was allowed for 12 days, which ultimately caused complete withering of the untreated control *A. stolonifera*, whereas the J6- and J10-treated *A. stolonifera* showed recovery of growth and development (Figure 3F). The bacterial density results for the drought stress experiment resembled the heat stress results (Figure 3G). After the recovery period, the total chlorophyll concentrations were 20 μg g^−1^ FW for both the J6- and J10-treated *A. stolonifera*, but no chlorophyll was detected in the control *A. stolonifera* (Figure 3H). The highest MDA concentration was found in the control *A. stolonifera* after both the drought stress period and the 12-day recovery period (Figure 3I). After the recovery period, the J6- and J10-treated *A. stolonifera* had significantly higher proline levels (16 mg g^−1^ FW) than the untreated control *A. stolonifera* (Figure 3J).

**Figure 4:**
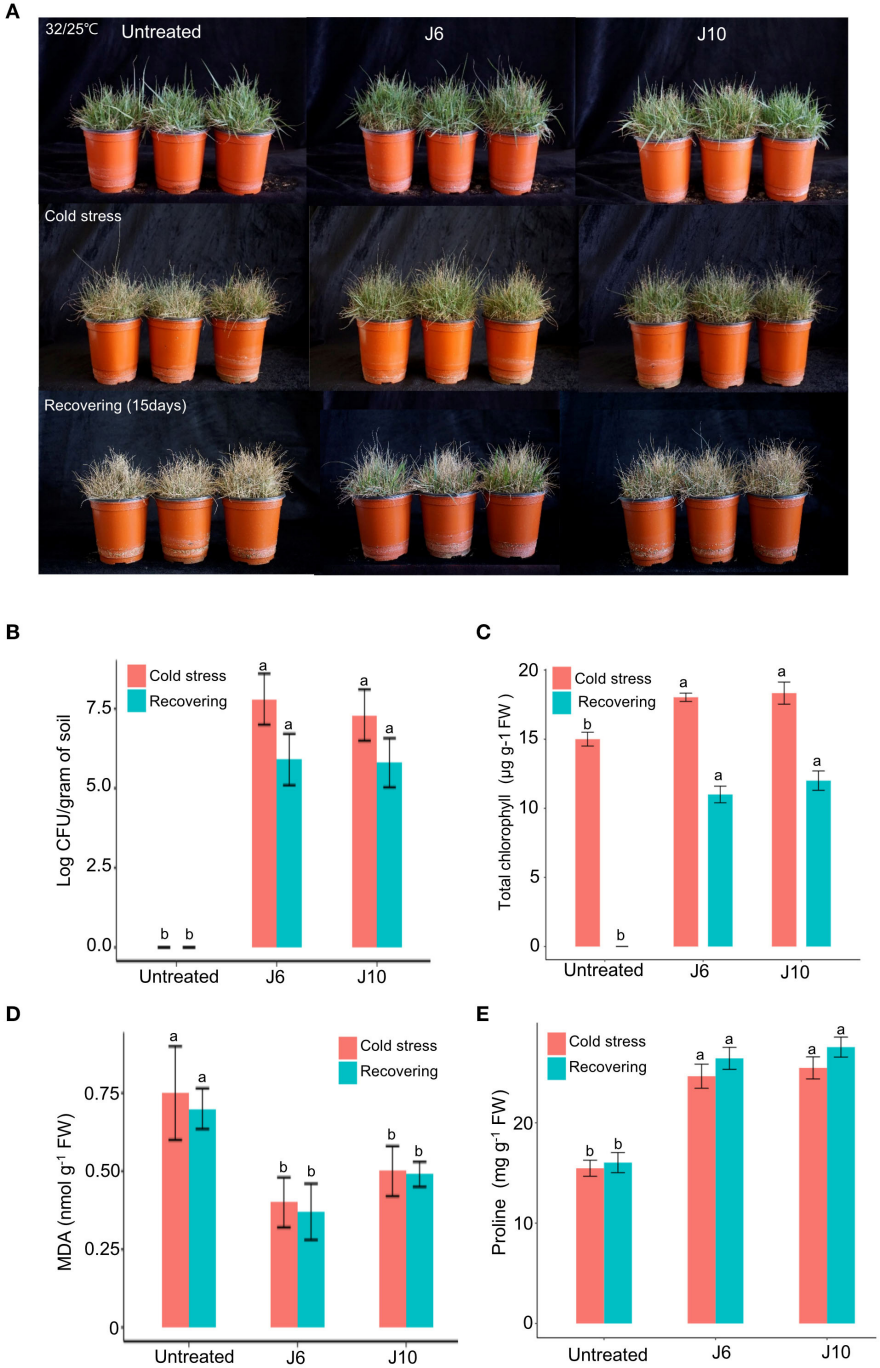
Alleviation of cold stress effects by *Streptomyces* sp. J6 and *Burkholderia vietnamiensis* J10 in *A. stolonifera*. **(A)** Turfgrass in various experimental treatment groups on completion of the 10-day application of cold stress and on completion of the 12-day recovery period. **(B)** Population density of J6 and J10 in turfgrass rhizospheres. **(C)** Total chlorophyll concentration. **(D)** Malondialdehyde (MDA) concentration. **(E)** Proline concentration. Different letters indicate a significant difference based on Duncan's multiple range test at *P* ≤ 0.05.

For warm-season turfgrass species, cold temperature is the most unfavorable abiotic stress. To evaluate whether the J6 and J10 strains can alleviate the effects of cold stress, a warm-season turfgrass cultivar (*Z. japonica*) was exposed to cold stress. The untreated control *Z. japonica* was found to have sustained more damage due to the cold stress than the J6- and J10-treated *Z. japonica*, which were relatively intact with regards to growth and development (Figure 4A). The trends regarding the population density of the J6 and J10 strains in the rhizosphere were identical to those indicated by the heat/drought stress results (Figure 4B). The total chlorophyll concentration in the J6- and J10-treated *Z. japonica* increased by 20.2 and 22.2%, respectively, compared to that in the control *Z. japonica*; after recovery, the total chlorophyll concentration was non-detectable, 11 and 12 μg g^−1^ FW in the untreated control, J6- and J10-treated *Z. japonica*, respectively (Figure 4C). The total chlorophyll, proline, and MDA concentrations exhibited similar outcomes in the cold-stress experiments (Figures 4C–E) to those in the heat/drought stress experiments. Thus, the total chlorophyll and proline concentrations were significantly increased in the J6- and J10-treated *Z. japonica* compared to the control *Z. japonica*, whereas the MDA concentration significantly decreased. The results of this study provide concrete evidence that the J6 and J10 strains have the potential to prevent large patch disease as well as the potential to reduce various abiotic stresses in turfgrass.

## Discussion

Turfgrasses can be grouped into cool-season (C3) and warm-season (C4) grasses, with C4 being more efficient at photosynthesis at high temperature and more tolerant to drought, and thus being preferred in warm and arid regions. Owing to these properties, warm-season turfgrasses (*Z. japonica*) are currently cultivated on a large scale worldwide, including South Korea. Various soil-borne fungal pathogens cause problematic diseases in turfgrass production. Among them large patch disease, caused by *R. solani* AG2-2, is the most devastating disease. Density of the large patch pathogen was detected as the highest in 13-year field, but in 2- and 25-years soils, the pathogen density was significantly lower than the 13-year soil. Interestingly, 2- and 25-years soil had similar microbial community structures compared to the 13-year soil. This finding suggests that soil microbiota in different turfgrass monoculture duration may influence pathogen density. Additionally, the most dominant bacteria were *Streptomycetaceae* and *Burkhoderiaceae* in 2- and 25-year soil, respectively. Obtained using a culture-dependent approach, selected antifungal bacteria *Streptomyces* sp. J6 and *B. vietnamiensis* J10 strain showed 100% identity of the most abundant *Streptomycetaceae* OTU16 and *Burkhoderiaceae* OTU866. Taken together, these results enabled the choice of *Streptomyces* sp. J6 and *B. vietnamiensis* J10 as functionally active bacteria for large patch disease suppression.

Much focus is being placed on plant-associated microbiomes due to the ability of microbes to improve crop productivity while also ameliorating external stresses (Mayak et al., 2004; Glick et al., 2007; Marulanda et al., 2009; Yang et al., 2009). Plant-microbe interactions related to disease have been frequently investigated, it is now becoming evident that the outcome of these interactions often involve plant-associated symbionts, which can disease suppressive effects (Mitter et al., 2016; Pieterse et al., 2016; Sánchez-Cañizares et al., 2017). However, the identification of functional microbes in the microbiome community of a plant with a history of long-term monoculture is rare. Wheat with take-all decline (TAD) is considered the best model for researching microbiomes associated with long-term monoculture (Weller, 2007). To biologically control TAD the control organism needs to be present in a minimal density (10^5^ cfu/g of soil) on the rhizosphere (Raaijmakers and Weller, 1998). Both *Streptomyces* sp. J6 and *B. vietnamiensis* J10 strains exhibited colonization ability on turfgrass rhizosphere up to 10^6^ cfu/g of soil. As results, the strains exhibited effective suppression of the large patch disease. This finding suggests that to identify effective microbes, analyses of microbiome communities and screening for functional microbes should be performed in agricultural fields with the specific plant species of interest.

*Rhizoctonia solani* produces disease symptoms by invading the laminae and ocreae of warm-season turfgrass (*Z. japonica*) and cold-season turfgrass (*A. stolonifera*) (Couch et al., 1990). This invasion of the turfgrass and the subsequent disease symptoms cause oxidative stress, which is then believed to increase the MDA concentration by causing lipid peroxidation initiated by peroxidation produced by intracellular structures due to cell membrane damage. Turfgrass are not only threatened by diseases but also various abiotic stresses. Upon exposure to stress, plants sustain oxidative damage such as lipid peroxidation caused by reactive oxygen species (Scandalios, 1993). Plants are especially vulnerable to damage caused by singlet oxygen and hydroxyl radicals, which are the byproducts of the oxidation of unsaturated fatty acids that comprise the cell wall. Ultimately, cells produce lipid hydroperoxides, which in turn detrimentally affect the dynamics of the cell membrane, causing secondary damage to cell wall proteins by facilitating the leakage of electrolytes from the inside (Moller et al., 2007). One of the most vital osmolytes is proline, whose concentration increases in plants undergoing abiotic stress (Farooq et al., 2009; Huang et al., 2014). In addition to making contributions to osmotic regulation, proline offers stability to sub-cellular structures such as proteins and membranes, neutralizes free radicals, and acts as a buffer to generate cellular redox potential (Ashraf and Foolad, 2007; Hayat et al., 2012). When plants are combatting abiotic stress, proline content is increased (Zarattini and Forlani, 2017). Inoculation with the J6 and J10 strains gave rise to an elevation in proline concentrations in turfgrass, which concurs with findings in maize (Naseem and Bano, 2014), sorghum (Grover et al., 2014), potato plants (Gururani et al., 2013), mung bean (Sarma and Saikia, 2014), and Arabidopsis (Cohen et al., 2015). The J6- and J10-treated *A. stolonifera* and *Z. japonica* increased tolerance against heat, drought, and cold stresses when compared to the un-inoculated control. Even though the biotic and abiotic stress mitigation properties of these strains is clear, the ability of strains J6 and J10 and co-inoculation on plant growth promotion still needs to be investigated to further expand the applicability of these bacteria in plant cultivation. Identifying more functional microbial strains with the potential to reduce both biotic and abiotic stresses in plants would serve as a sustainable tool for crop protection and management.

## Materials and Methods

### Investigation Sampling Sites and Assessment of the Properties of the Soil and Plant Samples

The turfgrass investigation was performed in Jangseong-gun, Republic of Korea. Assessments were conducted on three occasions in May, July, and September of 2015 (2-year field: N35°11, E126°40; 13-year field: N35°11, E126° 40; 25-year field: N35°11, E126 ^o^40). This sampling was carried out based on the times of the year in which large patch disease affects the warm-season turfgrass *Zoysia japonica* (zoysiagrass) in South Korea and when it is in remission (May: onset of disease; July: full-blown disease; September: remission). Samples collected from outside of the patches (depth between 5 and 25 cm from surface) in May, July, and September that had undergone 2, 13, and 25 years of monoculture were used for the study, with only samples collected in July being used to assess of the population density of the large patch pathogen, *R. solani*.

To assess the soil properties of the three fields in question, six locations per field were randomly selected and 3–5 cm of topsoil from the 10-cm surface layer (which is the maximum depth for planting turfgrass) was obtained per location for analysis of the soil texture and physicochemical properties (Supplementary Tables 1, 2). The soil texture was analyzed using the Beretta et al. (2014) method, and the physicochemical properties were analyzed using the soil analysis method of the National Institute of Agricultural Sciences of the Rural Development Administration. The soil's physical properties were assessed using a 100-mL-capacity core sampler. To prevent moisture loss from each sample of soil, it was sealed in a container before analysis of its density, porosity, moisture content using the soil gravimetric method (Fonteno, 1996).

To assess the growth and development of turfgrass, the warm-season turfgrass (*Z. japonica*) was investigated. Turf samples of 15 × 15 cm (*n* = 3) were obtained to assess the fresh and dry weight of the plant's total length, creeping stems, and aboveground and underground sections and to assess the aboveground population (which was measured by counting the number of stems within the 15 × 15 cm area) and overall length of the creeping stems. Before assessing the plant's dry weight, it was dried at 80°C for 72 h in a dryer (Model DS-80-5, Dasol Scientific Co., Ltd., Gyeonggido, Korea).

### Pyrosequencing of Rhizosphere Samples

To obtain the rhizosphere soil, a cup hole cutter was used to vigorously shake off excess soil on the turfgrass and a brush was used to collect the soil left behind on the roots of the turfgrass. Rhizosphere samples were collected from three turfgrass fields in which *Z. japonica* had been continuously cultivated for 2, 13, and 25 years, respectively. Sample collection involved using a cup hole cutter with a cutting diameter of 107 mm and a depth of 127 mm, with three replications (in May, July, and September) for each field (*n* = 3 per field).

To analyze the microbial communities, DNA was extracted using a FastDNA® SPIN Kit (Bio 101, MP Biomedicals, Irvine, CA, USA). This DNA was then quantified using a Nanodrop 2000c spectrophotometer (Thermo Scientific, Waltham, MA, USA). The DNA concentration ranged from 160 to 180 ng/μL and the mean A260/A280 ratio ranged from 1.98 to 2.02. The soil DNA from the rhizospheres collected in May, July, and September from each field (*n* = 3 per field) was pooled together to perform pyrosequencing. The V1–V3 region in the total DNA (100 ng/μL) was amplified using 27 mF (5′-GAGTTTGATCMTGGCTCAG-3′) and 518R (5′-WTTACCGCGGCTGCTGG-3′) primer pairs. The resulting product was outsourced to Macrogen Inc. (Seoul, Republic of Korea) for pyrosequencing using a 454 GS-FLX Titanium System with Roche Genome Sequencer (GS) FLX software (version 3.0) (Supplementary Table 5). The raw sequencing reads (FASTQ format) of low quality (mean Phred quality score >20) were removed. Additionally, the barcode tags of the filtered files were removed for both the forward and reverse sequences and the forward and reverse read lengths were 300 and 280 bp, respectively. The two sequences were paired to give total lengths of 400–420 bp. The Silva database version 132 (Quast et al., 2012) was employed to assign taxonomic data to the bacterial OTUs obtained. The similarities regarding the 16S rRNA nucleotide sequences were determined based on a similarity threshold of >97% and a relative abundance cutoff of >0.2%. Pyrosequencing analyses were carried out using the R package phyloseq version 1.22.3 (McMurdie and Holmes, 2013) and DEseq2 version 1.22.2 (Love et al., 2014), and the resulting data were graphed using ggplot2 version 3.1.0 (Wickham, 2016). The similarities regarding the 16S rRNA nucleotide sequences were determined based on the following similarity levels in the context of taxonomy: phylum, >75%; class, >80%; order, >85%; family, >90%; genus, >94%, and species, >97%. Heatmaps displaying clustering was conducted with the R package (superheat, http://github.com/rlbarter/superheat).

### Assessment of the Population Density of the Large Patch Pathogen *R. solani* in Rhizosphere Soil Using Quantitative PCR (qPCR)

To determine the population density of *R. solani*, which is the main cause of damage sustained in turfgrass rhizospheres, a series of qPCR analyses were performed on the rhizosphere samples of *Z. japonica* from the 2-, 13-, and 25-year fields. For this experiment, all samples were collected in July as the proliferation of *R. solani* is highest in July. Soil was collected from each of the three fields on three occasions. The soil samples from each field were blended to yield 1 g of consistent soil per field (2, 13, and 25-year fields) to be used for DNA extraction using a FastDNA® SPIN Kit (MP Biomedicals) following the accompanying instructions. The DNA was then subjected to ethanol precipitation and then used in qPCR (CFX Connect System, Bio-Rad, Hercules, CA, USA). The qPCR was performed using a 50 μL solution containing SYBR Green 25 μL (QPK-201T, Toyobo, Osaka, Japan), soil DNA 5 μL (100 ng/μL), primers (AG 2-2_F,R) 2 μL, and ddH_2_O 16 μL. The primers were from an *R. solani* AG-2-2-specific primer set (Toda et al., 2004). The qPCR involved 40 cycles, each of which consisted of 5 min at 95°C, 15 s at 95°C, 1 min at 60°C, and a further 1 min at 72°C sequentially. Upon completion of the reaction, the population density of the fungal pathogen was assessed by comparing the cycle threshold (CT) values and a standard curve. To create the standard curve, fungal genomic DNA was extracted using the cetyl trimethylammonium bromide (CTAB) technique (Porebski et al., 1997); *R. solani* AG-2-2 (IV) (KACC no. 40132) was supplied by the National Agrobiodiversity Center of the Rural Development Administration of the Republic of Korea. The extracted DNA underwent serial 10-fold dilution from 500 to 0.005 ng/μL to generate the standard curve using a NanoDrop 2000c spectrophotometer.

### Isolation and Antifungal Activity of Bacteria in the Rhizospheres

To isolate rhizosphere bacteria in order to select strains with antifungal activity, samples of turfgrass rhizosphere (*n* = 3 per field) were collected. These soil samples (1 g) were diluted to a dilution rate of 10^−1^–10^−8^ using distilled water and then smeared onto tryptic soy agar (TSA) medium [tryptic soy broth (TSB; BD, Franklin Lakes, NJ, USA) 30 g/L, agar 20 g/L] for culturing at 28°C. After 10 days, a single colony from each sample was inoculated in 100 μL of TSB in 96-well plates, which were shaken at 28°C for 2 days. Thereafter, 100 μL of 50% glycerol was added and then a Platemax® Pierceable Aluminum Heat Sealing Film (Axygen, Union City, CA, USA) was applied before storage at −80°C. To assess the antifungal activity of the bacteria, a three-step screening procedure was used. (1) The first step involved culturing the bacteria that had been prepared previously (and stored in 96-well plates at −80°C). This was done using OmniTrays (Sigma-Aldrich, St. Louis, MI, USA) containing PDK medium [potato dextrose (Difco) 10 g/L, peptone 10 g/L, agar 20 g/L] and culturing at 28°C for 3 days. *Rhizoctonia solani* mycelia (which had been obtained using a 0.5-mm cork borer and cultured on PDA medium) were placed between the bacterial colonies on the OmniTrays so that the antifungal activity of the bacteria could be assessed. (2) Next, 10 μL of the bacterial stains that had been shown to exhibit antifungal activity in the first step was suspended in sterile water (10^6^ cfu/mL) and streaked in a 3-cm line on PDK medium. After 3 days, *R. solani* mycelia (obtained using the 0.5-mm cork borer) were positioned in the center of the PDK plates, 2 cm away from the 3-cm line of bacteria. (3) The final step first involved selecting the bacteria that had demonstrated antifungal activity in the second step. Next, these bacterial strains were cultured until they reached an absorbance (at a wavelength of 595 nm) of 1.2 (~10^9^ cfu/mL upon measurement). Thereafter, 8-mm sterilized filter disks were placed on one side of the dish containing PDK medium on which 10 μL of the cultured bacteria was dropped for an additional 3 days of culturing. To explore the range of antifungal activity of the bacterial strains, *R. solani* mycelia or the major fungal pathogen responsible for summer patch, dollar spot, or spring dead spot pathogens (obtained using the 0.5-mm cork borer) were placed on the other side of the dish containing PDK medium and left for 3 days. The screening was repeated three times using the selected bacterial strains that exhibited antifungal activity (defined based on the distance between the bacteria and fungal pathogen after 3 days). The findings were classed as no inhibition; low inhibition, 0.1–0.5 cm; medium inhibition, 0.5–1 cm; strong inhibition, 1–1.5 cm; and the highest inhibition, >1.5 cm.

To establish the taxonomy of the two selected exceptional antifungal bacterial strains (*Streptomyces* sp. J6 and *B. vietnamiensis* J10), they were cultured on PDK medium at 30°C for 7 days and cells were then harvested into a 1.5-mL tube for DNA extraction using the CTAB method. The taxonomy was determined using 16S rRNA sequences, and 27F (5′-AGAGTTTGATCCTGGCTCAG-3′) and 1492R (5′-GGTTACCTTGTTACGACTT-3′) primers were employed to amplify the applicable domains while conforming to PCR guidelines. The amplified product was subjected to electrophoresis on 1% agarose gel to produce the necessary bands of DNA sequences. Sequencing was carried out by Macrogen Inc. The analytical data were then utilized to perform phylogenetic analyses using MEGA7 based on the National Center for Biotechnology Information (NCBI) GenBank BLAST algorithm and a maximum likelihood approach.

### Insertion of a Hygromycin-Resistance Gene in the *Streptomyces* sp. J6 Strain

A hygromycin-resistance gene was inserted using the modified version of the method proposed by Kieser et al. (2000). The *Streptomyces* sp. J6 strain was cultured on MS medium (mannitol 20 g/L, soya 20 g/L, agar 20 g/L) at 27°C for 7 days to allow it to form spores. Glycerol (20%) was then used to collect the spores by creating a suspension and the spores were filtered using syringes to produce a J6 spore suspension, which was stored at −20°C before use. *E. coli* ET12567 (pUZ8002), into which the pIJ10257 vector (Novotna et al., 2012) was inserted, was cultured on Luria-Bertani (LB) medium (25 g, agar 20 g/L) containing hygromycin (80 μg/mL). A single *E. coli* ET12567 colony was selected and cultured in LB liquid medium at 37°C for 9 h. Next, the J6 spore suspension (300 μL) was mixed with 500 μL 2xYT medium (1.6% tryptone, 1% yeast extract, 0.5% NaCl, pH 7) at 50°C for 10 min. Thereafter, 250 μL of the *E. coli* ET12567 strain was added and the solution was mixed to facilitate conjugation over the next 30 min. Upon completion of the conjugation reaction, the J6 strain was smeared on to MS medium containing hygromycin (80 μg/mL) and then cultured at 27°C for 4 days to obtain a colony with the hygromycin-resistance gene. To confirm the insertion of the hygromycin-resistance gene, the DNA of the colony was extracted using the CTAB method and amplified using Hyg det3 (5′-TCCGCTGTGACACAAGAATC-3′) and Hyg det5 (5′-CGGCTCATCACCAGGT AGG-3′) primers. The PCR involved 30 cycles, in which each cycle consisted of inducing a thermal modification reaction at 98°C for 3 min, denaturation at 98°C for 30 s, annealing at 55°C for 45 s, and extension at 72°C for 1 min. The final stage involved another 10 min at 72°C. Upon completion of the PCR, the insertion of the hygromycin-resistance gene was confirmed by electrophoresis. The inhibition of large patch disease and rhizosphere colonization ability of this hygromycin-resistant *Streptomyces* sp. J6 strain was then assessed, as described in later sections.

### Rifampicin-Resistant *Burkholderia vietnamiensis* J10 Strain

Isolation of spontaneous rifampicin-resistant (Rif^r^) mutants of the *B. vietnamiensis* J10 strain was carried out by transferring the colonies to LB medium containing rifampicin at a range of concentrations (50, 100, 150, and 200 μg/mL). The stability of rifampicin resistance was assessed by sub-culturing the Rif^r^ mutants on LB medium at 27°C for 48 h for 15 rounds and, after each round, comparing the CFUs (after diluting the bacterial suspension) by plating the solution on LB medium with and without rifampicin (200 μg/mL). The Rif^r^ mutant J10 strains were stored at −80°C before a selected strain was assessed for its inhibition of large patch disease and rhizosphere colonization ability using pot assays, exactly as the hygromycin-resistant J6 strain was assessed, as described below.

### Strain Culture and Pathogen Inoculum Production

The selected hygromycin-resistant J6 strain and the selected rifampicin-resistant J10 strain were each streaked separately on PDK medium and cultured at 25°C for 5 days. For each strain, a single colony was then obtained, which was sub-cultured in 5 mL of PDK broth at 28°C and 120 rpm for 3 days. The sub-cultured stains were then used to inoculate 200 mL of PDK broth and cultured at 28°C and 120 rpm for 14 days. The cultured J6 and J10 strains were then diluted to 10^6^ cfu/m with 1% methyl cellulose solution (final concentration of 0.1%). Thereafter, 100 mL of the solution (10^6^ cfu/mL) was added to plastic pots (12 × 11.5 × 10 cm) containing turfgrass (*Z. japonica*) three times at an interval of 2 days. After 7 days, the pots were inoculated with the large patch disease pathogen, *R. solani*.

The production of the *R. solani* inoculum involved mixing 700 g of sand and 300 g of oatmeal in 150 mL of distilled water, which was then subjected to three rounds of sterilization. The resulting mixture was inoculated with *R. solani* (which had been cultured on PDA medium for 5 days) and cultured at 27°C for 3 weeks. Turfgrass plants in 5-g plastic pots that had been grown for 4 weeks since they were sown were inoculated with this inoculum (10^4^ cfu/g of soil).

### Rhizosphere Colonization Ability and Inhibition of Large Patch Disease

To assess the rhizosphere colonization ability of *Streptomyces* sp. J6 and *B. vietnamiensis* J10, the growth conditions of turfgrass (*Z. japonica*) were reproduced using 12-h light (32°C)/12-h dark (24°C) photoperiods with ~228 μmol m^−2^s^−1^ light intensity and 70–80% relative humidity (Jeon et al., 2019). A total of 100 g of bed soil was placed into a plastic pot in which ~30 seeds were sown to assess the turfgrass growth and development. Germination took place 10 days later and the turfgrass was allowed to grow for 6 weeks. A randomized complete block design was used, with 10 pots per treatment and four treatments. The treatments consisted of an untreated control, *R. solani* only, *R. solani* with J6, and *R. solani* with J10. To determine the rhizosphere colonization ability, the population density of the J6 and J10 strains in the turfgrass rhizospheres was assessed. The hygromycin-resistant J6 strain and the rifampicin-resistant J10 strain were used to treat the plants (*n* = 10 per treatment). After 4 weeks, 1 g of soil was collected from the rhizospheres and diluted to a dilution rate of 10^−6^ with 9 mL of sterile water. The population density was assessed based on the dilution plate method, which involved the use of selective PDK medium (with rifampicin 100 μg/mL or hygromycin 80 μg /mL). To assess the inhibition of large patch disease (*n* = 10 per treatment), disease severity was rated on a scale from 0 to 4; 0 represented 0–20%, 1 represented 21–40%, 2 represented 41–60%, 3 represented 61–80%, and 4 represented 81–100% (Jeon et al., 2019).

### Assessment of Alleviation of Abiotic Stresses

In the abiotic stress experiments, both *Z. japonica* and *A. stolonifera* were used; cold stress was applied to *Z. japonica*, while *A. stolonifera* was subjected to heat or drought stress. There were three treatment groups, consisting of an untreated control group and J6 and J10 treatment groups. First, the plants were inoculated with the applicable strains and were left to undergo 7 days of growth and development before the abiotic stresses were applied. The growth conditions of *Z. japonica* were reproduced using 12-h light (32°C)/12-h dark (24°C) photoperiods with a light intensity of ~228 μmol m^−2^s^−1^ and a relative humidity of 70–80% (Jeon et al., 2019), while the growth conditions of *A. stolonifera* were reproduced using 12-h light (20°C)/12-h dark (15°C) photoperiods (McCann and Huang, 2007) with a light intensity of ~450 μmolm^−2^s^−1^ and a relative humidity of 70–80%.

The heat stress conditions were reproduced using 12-h light (35°C)/12-h dark (25°C) photoperiods (Jiang and Huang, 2001; McCann and Huang, 2007) and 100 mL of water was supplied once every 2 days for 10 days. The drought stress conditions were reproduced using 12-h light (22°C)/12-h dark (13°C) photoperiods and no water was supplied for 10 days. The cold stress conditions were reproduced using 12-h light (10°C)/12-h dark (0°C) photoperiods and 100 mL of water was supplied once every 2 days for 10 days. The 10 days of abiotic stress were followed by recovery for 12 days. Samples were collected from all treatments upon completion of stress application and upon completion of the recovery period.

### Chlorophyll Concentration

To ascertain the change in the chlorophyll concentration after the application of stresses, leaf samples were collected. The chlorophyll concentration was calculated as per the method described by Lichtenthaler (1987), which involved homogenizing 0.05 g of turfgrass leaf and 100% acetone. The resulting mixture was centrifuged at 2,800 × g (5810R, Eppendorf, Hamburg, Germany) at 4°C for 10 min. The absorbance values (at wavelengths of 661.6 and 644.8 nm) of the supernatant were then assessed using a spectrophotometer (UV-1800, Shimadzu, Kyoto, Japan). The absorbance values were substituted into the following formulae to calculate the chlorophyll concentration: Chl a = (11.24 × A_661.6_) − (2.04 × A_644.8_), Chl b = (20.13 × A_644.8_) − (4.19 × A_661.6_), Chl a+b = (7.05 × A_661.6_) + (18.09 × A_644.8_).

### MDA and Proline Concentrations

The concentration of MDA indicates oxidations stress by causing lipid peroxidation. The MDA concentration was measured as per the method proposed by Heath and Packer (1968), which involves homogenizing 0.2 g of leaf and 5% trichloroacetic acid and centrifuging the mixture at 4°C for 20 min at 12,000 × g (5810R, Eppendorf). Thereafter, 0.6% thiobarbituric acid was added to 2 mL of the resulting supernatant and the mixture was boiled in a water bath of 80°C for 15 min. The extracted fluid was further centrifuged at 4°C for 10 min at 12,000 × g. A spectrophotometer (UV-1800, Shimadzu) was then used to assess the absorbance values of the supernatant at wavelengths of 450, 532, and 600 nm. These values were then substituted into the following formula to calculate the MDA concentration: MDA (nmol^−1^) = 6.45 × (A_532_–A_600_)−0.56 × A_450_.

The proline concentration was measured by fragmenting 0.2 g of leaf in 10 mL of 3% sulfosalicylic acid, which was centrifuged (12,000 × g, 4°C, 20 min). Thereafter, 2 mL of the supernatant, 2 mL of acid-ninhydrin (glacial acetic acid 30 mL, 6 M phosphoric acid 20 mL, 1.25 g ninhydrin), and 2 mL of acetic acid were reacted together at 100°C for 1 h. Upon completion of the reaction, the solution was transferred to a container of ice. Next, 4 mL of toluene was added, and the mixture was vortexed for 15–20 s. The chromophore obtained using the toluene was separated from the other layers. The absorbance value (at a wavelength of 520 nm) was assessed using a spectrophotometer, with toluene being used as the blank. Finally, a standard curve was created using L-proline and quantitative analyses were carried out.

### Statistical Analysis

All data except for the sequence analysis data were analyzed using SigmaPlot version 11.0 (Systat Software Inc., Richmond, CA, USA). Statistical analyses of the mean values from each experiment were performed using ANOVA with Duncan's multiple range test. Statistical significance was defined as *P* ≤ 0.05. Graphs were plotted using ggplot2 version 3.1.0.

## Data Availability Statement

Sequencing data have been deposited in GenBank and all accession information is presented in Supplementary Table 5.

## Author Contributions

C-WJ, D-RK, and E-JB conducted abiotic stresses assays, collected, analyzed field samples, and soil. C-WJ, D-RK, and Y-SK performed pyrosequencing analyses and wrote the manuscript. All authors contributed to the article and approved the submitted version.

## Conflict of Interest

The authors declare that the research was conducted in the absence of any commercial or financial relationships that could be construed as a potential conflict of interest.

## References

[B1] Asano T. Senda M. Suga H. Kageyama K. (2010). Development of multiplex PCR to detect five *Pythium* species related to turfgrass diseases. J. Phytopathol. 158, 609–615. 10.1111/j.1439-0434.2009.01660.x

[B2] Ashraf M. Foolad M. R. (2007). Roles of glycine betaine and proline in improving plant abiotic stress resistance. Environ. Exp. Bot. 59, 206–216. 10.1016/j.envexpbot.2005.12.006

[B3] Beard J. B. (1972). Turfgrass: Science and Culture. Englewood Cliffs: Prentice-Hall.

[B4] Beretta A. N. Silbermann A. V. Paladino L. Torres D. Bassahun D. Musselli R. (2014). Soil texture analyses using a hydrometer: odification of the Bouyoucos method. Cienc. Investig. Agrari. 41, 263–271. 10.4067/S0718-16202014000200013

[B5] Cha J. Y. Han S. Hong H. J. Cho H. Kim D. Kwon Y.. (2016). Microbial and biochemical basis of a Fusarium wilt-suppressive soil. ISME J. 10, 119. 10.1038/ismej.2015.95 26057845 PMC4681868

[B6] Cohen A. Bottini R. Pontin M. Berli F. Moreno D. Boccanlandro H.. (2015). *Azospirillum brasilense* ameliorates the response of *Arabidopsis thaliana* to drought mainly via enhancement of ABA levels. Physiol. Plant. 153, 79–90. 10.1111/ppl.12221 24796562

[B7] Couch H. B. Lucas L. T. Haygood R. A. (1990). The nature and control of *Rhizoctonia* blight. Golf Course Manag. 58, 49–58.

[B8] Farooq M. Wahid A. Kobayashi N. Fujita D. Basra S. M. A. (2009). Plant drought stress: effects, mechanisms and management. Agron. Sustain. 29, 185–212. 10.1051/agro:2008021

[B9] Fonteno W. C. (1996). Growing media: types and physical/chemical properties, in Water, Media, and Nutrition for Greenhouse Crops Reed D. W, ed, (Batavia, IL: Ball Publishing), 93-122.

[B10] Glick B. R. Cheng Z. Czarny J. Duan J. (2007). Promotion of plant growth by ACC deaminase-producing soil bacteria. Eur. J. Plant Pathol. 119, 329–339. 10.1007/s10658-007-9162-416121231

[B11] Grover M. Madhubala R. Ali S. Z. Yadav S. K. Venkateswarlu B. (2014). Influence of Bacillus spp. strains on seedling growth and physiological parameters of sorghum under moisture stress conditions. J. Basic Microbiol. 54, 951–961. 10.1002/jobm.201300250 24027209

[B12] Gururani M. A. Upadhyaya C. P. Baskar V. Venkatesh J. Nookaraju A. Park S. W. (2013). Plant growth-promoting rhizobacteria enhance abiotic stress tolerance in *Solanum tuberosum* through inducing changes in the expression of ROS-scavenging enzymes and improved photosynthetic performance. J. Plant Growth Regul. 32, 245–258. 10.1007/s00344-012-9292-6

[B13] Hayat S. Hayat Q. Alyemeni M. N. Wani A. S. Pichtel J. Ahmad A. (2012). Role of proline under changing environments: a review. Plant Signal. Behav. 7, 1456–1466. 10.4161/psb.21949 22951402 PMC3548871

[B14] Heath R. L. Packer L. (1968). Photoperoxidation in isolated chloroplasts. I. Kinetics and stoichiometry of fatty acid peroxidation. Arch. Biochem. Biophys. 125:180–198. 10.1016/0003-9861(68)90654-1 5655425

[B15] Huang B. DaCosta M. Jiang Y. (2014). Research advances in mechanisms of turfgrass tolerance to abiotic stresses: from physiology to molecular biology. Crit. Rev. Plant Sci. 33, 141–189. 10.1080/07352689.2014.870411

[B16] Jeon C. W. Kim D. R. Kwak Y. S. (2019). Valinomycin, produced by *Streptomyces* sp. S8, a key antifungal metabolite in large patch disease suppressiveness. World J. Microbiol. Biotechnol. 35, 128. 10.1007/s11274-019-2704-z 31375920

[B17] Jiang Y. Huang B. (2001). Drought and heat stress injury to two cool-season turfgrasses in relation to antioxidant metabolism and lipid peroxidation. Crop Sci. 41, 436–442. 10.2135/cropsci2001.412436x

[B18] Jones D. L. Nguyen C. Finlay R. D. (2009). Carbon flow in the rhizosphere: carbon trading at the soil-root interface. Plant Soil. 321, 5–33. 10.1007/s11104-009-9925-0

[B19] Kieser T. Bibb M. Chater K. Butter M. Hopwood D. Bittner M. (2000). Practical Streptomyces Genetics: A Laboratory Manual. Norwich, UK: John Innes Centre.

[B20] Levy A. Gonzalez I. S. Mittelviefhaus M. Clingenpeel S. Paredes S. H. Miao J.. (2018). Genomic features of bacterial adaptation to plants. Nat. Genetics 50, 138–150. 10.1038/s41588-017-0012-9 29255260 PMC5957079

[B21] Lichtenthaler H. (1987). Chlorophyll and carotenoids: pigments of photosynthetic biomembranes. Meth. Enzymol. 148, 350–382. 10.1016/0076-6879(87)48036-1

[B22] Love M. I. Huber W. Anders S. (2014). Moderated estimation of fold change and dispersion for RNA-seq data with DESeq2. Genome Biol. 15:550. 10.1186/s13059-014-0550-8 25516281 PMC4302049

[B23] Marasco R. Rolli E. Fusi M. Michoud G. Daffonchio D. (2018). Grapevine rootstocks shape underground bacterial microbiome and networking but not potential functionality. Microbiome 6:3 10.1186/s40168-017-0391-2 29298729 PMC5751889

[B24] Marulanda A. Barea J.-M. Azcón R. (2009). Stimulation of plant growth and drought tolerance by native microorganisms (AM fungi and bacteria) from dry environments: mechanisms related to bacterial effectiveness. J. Plant Growth Regul. 28, 115–124. 10.1007/s00344-009-9079-6

[B25] Mayak S. Tirosh T. Glick B. R. (2004). Plant growth-promoting bacteria that confer resistance to water stress in tomatoes and peppers. Plant Sci. 166, 525–530. 10.1016/j.plantsci.2003.10.025

[B26] McCann S. E. Huang B. (2007). Effects of trinexapac-ethyl foliar application on creeping bentgrass responses to combined drought and heat stress. Crop Sci. 47, 2121–2128. 10.2135/cropsci2006.09.0614

[B27] McMurdie P. J. Holmes S. (2013). phyloseq: an R package for reproducible interactive analysis and graphics of microbiome census data. PLoS ONE 8:e61217. 10.1371/journal.pone.0061217 23630581 PMC3632530

[B28] Mitter B. Pfaffenbichler N. Sessitsch A. (2016). Plant–microbe partnerships in 2020. Microb. Biotechnol. 9, 635–640. 10.1111/1751-7915.12382 27418200 PMC4993182

[B29] Moller I. M. Jensen P. E. Hansson A. (2007). Oxidative modifications to cellular components in plants. Annu. Rev. Plant Biol. 58, 459–481. 10.1146/annurev.arplant.58.032806.103946 17288534

[B30] Naseem H. Bano A. (2014). Role of plant growth-promoting rhizobacteria and their exopolysaccharide in drought tolerance in maize. J. Plant Interact. 9, 689–701. 10.1080/17429145.2014.902125

[B31] Novotna G. Hill C. Vincent K. Liu C. Hong H. J. (2012). A novel membrane protein, VanJ, conferring resistance to teicoplanin. Antimicrob. Agents Chemother. 56, 1784–1796. 10.1128/AAC.05869-11 22232274 PMC3318380

[B32] Obasa K. Fry J. Kennelly M. (2012). Susceptibility of zoysiagrass germplasm to large patch caused by *Rhizoctonia solani*. HortScience 47, 1252–1256. 10.21273/HORTSCI.47.9.125230722516

[B33] Pieterse C. M. de Jonge R. Berendsen R. L. (2016). The soil-borne supremacy. Trends Plant Sci. 21, 171–173. 10.1016/j.tplants.2016.01.018 26853594

[B34] Porebski S. Bailey L. G. Baum R. (1997). Modification of a CTAB DNA extraction protocol for plants containing high polysaccharide and polyphenol components. Plant Mol. Biol. Rep. 15, 8–15. 10.1007/bf02772108

[B35] Poudel R. Jumpponen A. Kennelly M. M. Rivard C. L. Gomez-Montano L. Garrett K. A. (2019). Rootstocks shape the rhizobiome: rhizosphere and endosphere bacterial communities in the grafted tomato system. Appl. Environ. Microb. 85, e01765–e01718. 10.1128/AEM.01765-18 30413478 PMC6328775

[B36] Quast C. Pruesse E. Yilmaz P. Gerken J. Schweer T. Yarza P.. (2012). The SILVA ribosomal RNA gene database project: improved data processing and web-based tools. Nucleic Acids Res. 41, D590–D59. 10.1093/nar/gks1219 23193283 PMC3531112

[B37] Raaijmakers J. M. Weller D. M. (1998). Natural plant protection by 2,4-diacetylphloroglucinol-producing *Pseudomonas* spp. in take-all decline soils. Mol. Plant Microbe Interact. 11, 144–152. 10.1094/MPMI.1998.11.2.144

[B38] Raghothama K. G. Karthikeyan A. S. (2005). Phosphate acquisition. Plant Soil. 274, 37–49. 10.1007/s11104-004-2005-6

[B39] Richardson A. E. (2001). Prospects for using soil microorganisms to improve the acquisition of phosphorus by plants. Aust. J. Plant Physiol. 28, 897–906. 10.1071/PP01093

[B40] Sánchez-Cañizares C. Jorrín B. Poole P. S. Tkacz A. (2017). Understanding the holobiont: the interdependence of plants and their microbiome. Curr. Opin. Microbiol. 38, 188–196. 10.1016/j.mib.2017.07.001 28732267

[B41] Sarma R. Saikia R. (2014). Alleviation of drought stress in mung bean by strain *Pseudomonas aeruginosa* GGRJ21. Plant Soil. 377, 111–126. 10.1007/s11104-013-1981-9

[B42] Scandalios J. G. (1993). Oxygen stress and superoxide dismutases. Plant Physiol. 101, 7–12. 10.1104/pp.101.1.7 12231660 PMC158641

[B43] Simmons M. Nertelsen M. Windhager S. Zafian H. (2011). The performance of native and non-native turfgrass monocultures and native turfgrass polycultures: an ecological approach to sustainable lawns. Ecol. Eng. 37, 1095–1103. 10.1016/j.ecoleng.2011.03.004

[B44] Smiley R. W. Dernoeden P. H. Clarke B. B. (2005). Compendium of Turfgrass Diseases, 3rd ed. St. Paul, MN: American Phytopathological Society 10.1094/9780890546154

[B45] Tang M.-J. Zhu Q. Zhang F.-M. Zhang W. Yuan J. Sun K.. (2019). Enhanced nitrogen and phosphorus activation with an optimized bacterial community by endophytic fungus *Phomopsis liquidambari* in paddy soil. Microbiol Res. 221, 50–59. 10.1016/j.micres.2019.02.005 30825941

[B46] Toda T. Mushika T. Hyakumachi M. (2004). Development of specific PCR primers for the detection of *Rhizoctonia solani* AG 2-2 LP from the leaf sheaths exhibiting large-patch symptom on Zoysia grass. FEMS Microbiol. Lett. 232, 67–74. 10.1016/S0378-1097(04)00016-3 15019736

[B47] van Bruggen A. H. C Arneson P. A. (1984). Resistance in *Rhizoctonia solani* to tolclofos-methyl. Neth. J. Plant Pathol. 90, 95–106. 10.1007/BF01994514

[B48] Vincelli P. Munshaw G. (2014). Chemical Control of Turfgrass Diseases. Agriculture and Natural Resources Publications. University of Kentucky. Available online at: http://uknowledge.uky.edu/cgi/viewcontent.cgi?article=1021&context=anr_reports

[B49] Weller D. M. (2007). Pseudomonas biocontrol agents of soilborne pathogens: looking back over 30 years. Phytopathology 97, 250–260. 10.1094/PHYTO-97-2-0250 18944383

[B50] Wickham H. (2016). ggplot2: Elegant Graphics for Data Analysis. 2nd ed New York, NY: Springer International Publishing.

[B51] Yang J. Kloepper J. W. Ryu C. M. (2009). Rhizosphere bacteria help plants tolerate abiotic stress. Trends Plant Sci. 14, 1–4. 10.1016/j.tplants.2008.10.004 19056309

[B52] Zarattini M. Forlani G. (2017). Toward unveiling the mechanisms for transcriptional regulation of proline biosynthesis in the plant cell response to biotic and abiotic stress conditions. Front. Plant Sci. 8:927. 10.3389/fpls.2017.00927 28626464 PMC5454058

[B53] Zhalnina K. Louie K. B. Hao Z. Mansoori N. da Rocha U. N. Shi S.. (2018). Dynamic root exudate chemistry and microbial substrate preferences drive patterns in rhizosphere microbial community assembly. Nat. Microbiol. 3, 470–480. 10.1038/s41564-018-0129-3 29556109

[B54] Zhang J. Fu Y. Xiang W. (2019). Antifungal, plant growth-promoting and genomic properties of an endophytic actinobacterium *Streptomyces* sp. NEAU-S7GS2. Front. Microbiol. 10:2077. 10.3389/fmicb.2019.02077 31551997 PMC6746918

