# Supplementary material for: Changes in Bacterial Community Structure and Enriched Functional Bacteria Associated With Turfgrass Monoculture
Source: Front Bioeng Biotechnol. 2021 Jan 15;8:530067. doi: 10.3389/fbioe.2020.530067 (PMC7844203; doi:10.3389/fbioe.2020.530067)
Supplement: Supplementary file 1 [file Table_1.DOCX]

**Supplementary Table 1.** Soil physical characteristics of turfgrass cultivation fields

| Year of monoculture | Soil texture | Bulk density (g·m^-3^) | Porosity (%) | Water content (%) | Three phases (%) | | |
| --- | --- | --- | --- | --- | --- | --- | --- |
|  |  |  |  |  | Solid | Liquid | Gaseous |
| 2Y | Loam | 1.58±0.05a^z^ | 40.4±1.7a | 13.5±0.2b | 59.6±1.7a | 22.3±0.8a | 19.1±2.5a |
| 13Y | Sandy loam | 1.54±0.03a | 41.9±1.2a | 12.3±0.3b | 58.1±1.2a | 23.9±0.7a | 20.0±1.8a |
| 25Y | Sandy loam | 1.68±0.02a | 36.8±0.7b | 17.3±0.5a | 60.2±0.7a | 22.0±1.1a | 19.7±0.7a |

^z^Mean separation within columns by Duncan’s multiple range test, *P* ≤ 0.05. Perform 3 replications for each sampling (n=3).

**Supplementary Table 2.** Soil chemical characteristics of turfgrass cultivation fields

| Year of monoculture | pH  (1:5) | EC  (dS·m^-1^) | T-N^y^  (g·kg^-1^) | O.M.  (g·kg^-1^) | Av. P_2_O_5_  (mg·kg^-1^) | Av. SiO_2_  (mg·kg^-1^) | Three phases(%) | | |
| --- | --- | --- | --- | --- | --- | --- | --- | --- | --- |
|  |  |  |  |  |  |  | Solid | Liquid | Gaseous |
| 2Y | 6.1a^z^ | 1.48a | 1.4a | 9.0a | 160.0a | 45.0a | 0.22a | 2.94a | 1.48a |
| 13Y | 5.4b | 1.46a | 1.5a | 9.0a | 158.0a | 41.0a | 0.27a | 2.03a | 1.74a |
| 25Y | 5.1b | 1.46a | 1.2a | 10.0a | 162.0a | 44.0a | 0.24a | 2.40a | 1.67a |

^y^T-N: total nitrogen; O.M.: organic matter; Av.P_2_O_5_: available P_2_O_5_; Av.SiO_2_: available SiO_2_; Ex. Cation: exchangeable cation.

^z^Mean separation within columns by Duncan’s multiple range test, *P* ≤ 0.05. Perform 3 replications for each sampling (n=3).

**Supplementary Table 3.** Number of sequencing read counts of the rhizosphere samples

| Sources | Sample | Number of reads | Total bases |
| --- | --- | --- | --- |
| Rhizosphere | 2Y-May | 15016 | 4,805,120 |
|  | 13Y-May | 13170 | 4,241,400 |
|  | 25Y-May | 13473 | 4,311,360 |
|  | 2Y-Jul | 14487 | 4,635,840 |
|  | 13Y-Jul | 11893 | 3,805,760 |
|  | 25Y-Jul | 13700 | 4,384,000 |
|  | 2Y-Sep | 16316 | 5,221,120 |
|  | 13Y-Sep | 14652 | 4,688,640 |
|  | 25Y-Sep | 15973 | 5,111,360 |

**Supplementary Table 4.** List of OTUs analysed in this study (separated excel file)

**Supplementary Table 5.** GenBank accession numbers for pyrosequencing

|  | 2Y-May | 2Y-Jul | 2Y-Seq | 13Y-May | 13Y-Jul | 13Y-Seq | 25Y-May | 25Y-Jul | 25Y-Seq |
| --- | --- | --- | --- | --- | --- | --- | --- | --- | --- |
| SAR | SRR11747659 | SRR11747660 | SRR11747661 | SRR11747662 | SRR11747663 | SRR11747664 | SRR11747665 | SRR11747666 | SRR11747667 |
| Bioproject | PRJNA630115 | PRJNA630116 | PRJNA630341 | PRJNA630343 | PRJNA630344 | PRJNA630345 | PRJNA630346 | PRJNA630347 | PRJNA630348 |
| Biosample | SAMN14825241 | SAMN14825242 | SAMN14832733 | SAMN14832750 | SAMN14832751 | SAMN14832752 | SAMN14832795 | SAMN14832796 | SAMN14832797 |

**Supplementary Table 6.** Antifungal range of *Streptomyces* sp. J6 and *Burkholderia vietnamiensis* J10

| Origin | Isolate | Large patch | Summer patch | Dollar spot | Spring dead spot |
| --- | --- | --- | --- | --- | --- |
| 2Y-Rhizosphere | J6 | ++++ | ++++ | ++++ | ++++ |
| 25Y-Rhizosphere | J10 | ++++ | ++++ | ++++ | ++++ |

**++++** indicated size of antagonistic clean zone more than 1-1.5 cm on PDK plate. Antifungal activity of the strains was performed three replication (*n* = 3)
